# Specific and Reversible Immobilization of Proteins Tagged to the Affinity Polypeptide C-LytA on Functionalized Graphite Electrodes

**DOI:** 10.1371/journal.pone.0087995

**Published:** 2014-01-31

**Authors:** Daniel Bello-Gil, Beatriz Maestro, Jennifer Fonseca, Juan M. Feliu, Víctor Climent, Jesús M. Sanz

**Affiliations:** 1 Instituto de Biología Molecular y Celular, Universidad Miguel Hernández, Elche, Spain; 2 Instituto Universitario de Electroquímica. Universidad de Alicante, Alicante, Spain; Northeastern University, United States of America

## Abstract

We have developed a general method for the specific and reversible immobilization of proteins fused to the choline-binding module C-LytA on functionalized graphite electrodes. Graphite electrode surfaces were modified by diazonium chemistry to introduce carboxylic groups that were subsequently used to anchor mixed self-assembled monolayers consisting of *N,N*-diethylethylenediamine groups, acting as choline analogs, and ethanolamine groups as spacers. The ability of the prepared electrodes to specifically bind C-LytA-tagged recombinant proteins was tested with a C-LytA-β-galactosidase fusion protein. The binding, activity and stability of the immobilized protein was evaluated by electrochemically monitoring the formation of an electroactive product in the enzymatic hydrolysis of the synthetic substrate 4-aminophenyl β-D-galactopyranoside. The hybrid protein was immobilized in an specific and reversible way, while retaining the catalytic activity. Moreover, these functionalized electrodes were shown to be highly stable and reusable. The method developed here can be envisaged as a general, immobilization procedure on the protein biosensor field.

## Introduction

The development of novel and gentle enzyme immobilization techniques is a subject of major relevance in Biotechnology, including enzymatic biosensor applications [1], [2]. Although the immobilization of enzymes has been widely shown to improve their stability and lengthen their half-life, allowing them to carry out its catalytic activity in a larger range of environments [3], it should be pointed out that the immobilization procedure leads in many cases to structural deformation due to multi-point attachment or to a poorer accesibility of the substrate to the active site, often leading to partial or total loss of activity [4].

To improve the performance and long-term stability of enzymatic sensors, one of the major challenges is to achieve a controlled binding of the protein to the electronic component, yet retaining their full activity and stability [2], [5], [6]. In this sense, biochemical affinity reactions offer a versatile strategy for protein immobilization, providing important advantages over other techniques, as these procedures combine a gentler approach, compared to the harsher (even denaturing) covalent methods, with the reduction of non-specific adsorption processes that characterize other physical, non-covalent methods [7]. This specific affinity interaction between proteins and uniform substrate surfaces may be used for the development of sensitive biosensors and other bioelectronic devices [1], [4], [8]–[10].

The functionalization of solid surfaces combined with the use of affinity polypeptide tags requires both the stable introduction of functional groups over the electrode surface by the use, for example, of self-assembled monolayers (SAM), and the genetic modification of the protein to introduce a specific affinity polypeptide tag that subsequently would bind to the functionalized surface [11].

Among the described affinity polypeptides, the hexahistidine tag (his-tag) is by far the most commonly used, not only for high-throughput protein purification [12], [13], but also for the immobilization of histidine-tagged proteins on electrode surfaces [11], [14], [15]. However, the use of this affinity system is not free of disadvantages, since specificity and stability of immobilized metal affinity chromatography (IMAC) has been shown not to be as high as with other affinity methods [1], [13], and the use of many reagents such as chelating and reducing agents, amines or anionic detergents is incompatible with this bioaffinity process [16]. Moreover, the use of the his-tag is not suitable for proteins containing metal ions as these may be chelated by the activated support [17]. Therefore, the development of alternative strategies is desirable.

One promising affinity tag for protein immobilization on electrodes is represented by the C-terminal domain of the LytA amidase from *Streptococcus pneumoniae* (C-LytA), a choline-binding protein (CBP) of the pneumococcal cell wall [18]. CBPs have specialized in the recognition of the choline residues present in the cell wall thanks to the fusion of different catalytic modules with the so-called choline-binding modules [19] (CBMs). The affinity of the C-LytA module for choline and structural analogues [20], [21], has allowed the development of an efficient system for the overexpression and purification of fusion proteins tagged to C-LytA using commercial resins such as diethylaminoethanol (DEAE) cellulose [22], [23] or even aqueous two-phase systems [24], and that has been commercialized (http://www.biomedal.com). Moreover, the crystal structure of the protein has been solved [25] and its folding and stability have been investigated [26] and modified by protein engineering [27]. Besides, the use of relatively sizeable tags such as C-LytA may help the immobilized protein of interest to avoid steric hindrances with the support and to increase its mobility [2]. The C-LytA tag has been succesfully employed to immobilize proteins onto gold electrodes [6]. In this work, a mixed SAM of thiol chains functionalized with choline was synthesized over a template of thiocarboxylic acid adsorbed onto gold, displaying specific affinity for β-galactosidase tagged to C-LytA (CLyt-βGal protein), and keeping the full β-galactosidase activity.

Besides gold, graphite is also considered a very convenient material as substrate in biosensor development due to their good electrochemical properties, ease-of-handling and low cost, and the wide exchange area that determines a higher amperometric response [28]. Moreover, the functionalization of graphitic substrates by electrochemical reduction of aryl diazonium salts enhances the appeal of this material, as it opens the chance of modifying the surface with a large range of groups with different chemical properties [29], [30]. In this sense, an interesting approach has been conducted by Blum and co-workers by direct electro-addressing of a modified antibody onto a screen-printed graphite electrode surface [31].

The present work is aimed to widen the application potential of the C-LytA tag for the generation of specific, stable and reversible enzymatic graphite electrodes, using the chimera CLyt-βGal as testing protein. The system constitutes a new efficient strategy for the immobilization of proteins with interest on molecular bioelectronics and biosensoring applications using stable and cost-effective functionalized graphite electrodes.

## Materials and Methods

### Purification of the CLyt-βGal protein

CLyt-βGal was purified by affinity chromatography as described by Sánchez-Puelles et al (1992) with minor modifications. Plasmid pEG40 [23], which allows the overexpression of the C-LytA-β-galactosidase hybrid gene, was purified using the QIAprep® Spin Miniprep Kit (QIAGEN) and used to transform freshly prepared *Escherichia coli* competent RB791 cells [32] using the protocol described by Lederberg and Cohen [33]. A single colony was used to inoculate Luria-Bertani medium (LB) supplemented with ampicillin (0.1 mg mL^−1^) to select for plasmid conferring resistance [34], and cultures were grown at 37°C until reaching an O.D._600 nm_ around 0.6. Expression of the CLyt-βGal gene was then induced by the addition of 0.5 mM isopropyl-β-D-thiogalactopyranoside (IPTG) to induce the lac promoter, and incubation proceeded overnight at 30°C. Cells were harvested by centrifugation, lysed by sonication and centrifuged again at 10 000 *g* for 10 minutes at 4°C to obtain a clear supernatant. The cell extracts were incubated at 4°C (1 h, mild orbital agitation) with 3 g of DEAE-cellulose (Sigma-Aldrich) equilibrated on 20 mM sodium phosphate pH 7.0. A chromatography column (30×1.5 cm) was packed with the suspension, washed extensively with buffer containing 20 mM sodium phosphate, pH 7.0 plus 1 M NaCl, and eluted with the same buffer containing 140 mM choline chloride (Sigma-Aldrich). Purity of the samples was checked by sodium dodecyl sulfate polyacrylamide gel electrophoresis [35]. The protein concentration was evaluated by absorption spectroscopy using a molar absorption coefficient at 280 nm of 329070 M^−1^ cm^−1^, calculated using the online software ProtParam from the Expasy toolbox (http://web.expasy.org/protparam).

### Electrode preparation

**Figure 1:**
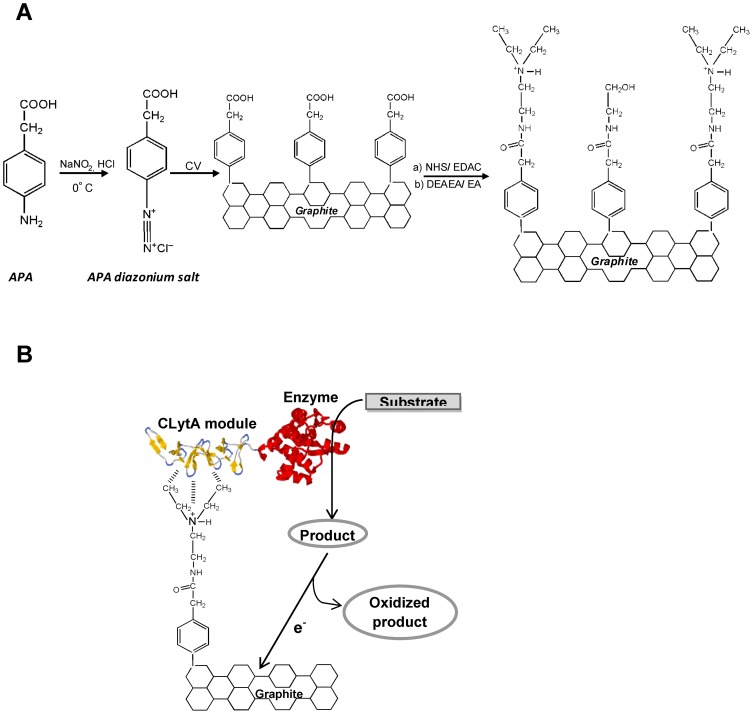
Schemes of functionalization of graphite electrodes for affinity immobilization of C-LytA-tagged proteins. (A) Sequence of chemical reactions involved in the generation of choline-like functionalized graphite electrodes. APA: 4-aminophenylacetic acid; CV, cyclic voltammetry. (B) Schematic illustration of an immobilized enzyme tagged to C-LytA onto the modified surface of a graphite electrode. The enzyme hydrolyzes the substrate yielding a product which is further oxidized on the electrode surface, producing an increase in positive current intensity that can be registered by cyclic voltammetry and chronoamperometry (not to scale, molecular details are not accurate).

Graphite rods (3 mm diameter, Grade 1 Spec-Pure; TED Pella, Inc., Reddong, CA) were polished using an abrasive disc P1200 (Buehler, Illinois, USA), sonicated in ultra-pure water for ten minutes, kept in 1 M NaOH for 30 minutes and then in 1 M HCl overnight. After each step, graphite rods were washed extensively with ultra-pure water. The functionalized working electrodes were prepared following the scheme in Figure 1A. For the diazotation of 4-aminophenylacetic acid (APA, Sigma-Aldrich), 956 µL of a 4.2 mM solution of the aromatic amine in 1 M aqueous HCl/ethanol 50∶50 (v/v) was stirred with 44 µL of an ice-cold 7.6 mg mL^−1^ solution sodium nitrite in water/ethanol 50∶50 for 5 min. Then, the solution was added to 20 mL of ice-cold 0.1 M aqueous HCl/ethanol 50∶50, resulting in a 0.19 mM solution of the diazonium salt [36]. The reductive adsorption of the salt onto the electrode was achieved by two scans of cyclic voltammetry (CV) [37] between 0.5 and −0.3 V at 50 mV s^−1^. Activation of the carboxylic groups was accomplished by rinsing the modified electrode sequentially with ethanol and ultra-pure water and then immersed for 3 h at 4°C in a dioxane solution of 0.1 M *N*-hydroxysuccinimide (NHS; Sigma-Aldrich) containing 0.1 M of 1-ethyl-3-[3-(dimethylamino) propyl]carbodiimide (EDAC; Sigma-Aldrich). Then, the electrode was rinsed three times with dioxane and air-dried [6]. Finally, the esterification of the activated carboxyl groups was carried out by dipping the electrode in a solution of 3.3 M ethanolamine (EA) and 0.41 M *N,N*-diethylethylenediamine (DEAEA) (8∶1 molar ratio) during 24 h at 4°C. Ethanolamine was used as a spacer between the DEAEA molecules which are the true attachment points for the protein, and to reduce any destabilizing electrostatic effects of the protonated tertiary amine at pH 7.0 [38]. The electrodes were finally washed with ultra-pure water and used immediately or stored at 4°C.

### Immobilization of CLyt-βGal onto the functionalized graphite electrodes

The functionalized electrodes were immersed in a 0.1 g L^−1^ solution of the CLyt-βGal protein in 20 mM sodium phosphate buffer pH 7.0 at 25°C for 15 min. Then, the electrodes were washed for 15 min with 20 mM sodium phosphate buffer pH 7.0 plus 0.1 M NaCl in order to release the non-specifically bound protein. Commercial β-galactosidase from *E. coli* (enzyme immunoassay quality; Sigma-Aldrich) was used as control of non-specific binding to the electrodes. To check the enzymatic activity, the electrode containing the adorbed enzyme was disposed in a meniscus configuration in relation to the solution, to make sure that the exposed area was the same in all the samples.

### Enzymatic activity colorimetric assay

β-Galactosidase activity was assayed spectrophotometrically, following at 420 nm the formation of *o*-nitrophenol (ε_420_ = 4500 M^−1^ cm^−1^) produced by the hydrolysis of 5 mM *o*-nitro-phenyl-β-D-galactopiranoside (ONPG) after a 10-minute incubation [39]. The reaction was stopped with Na_2_CO_3_ 0.12 M. One unit of enzymatic activity was defined as the amount of enzyme that produces 1 nmol of *o*-nitrophenol per minute at 28°C and pH 7.0.

### Electrochemical experiments

The electrochemical assays were performed using a conventional three-electrode cell. A platinum coiled wire was used as auxiliary electrode and an Ag/AgCl electrode in 3 M KCl, connected to the cell by a Luggin capillary filled with the same electrolyte solution, was used as reference electrode. The cell was connected to a μAutolab type III potentiostat controlled with GPES software. The electrolytes were prepared from suprapur chemical reagents (Merck) in ultra-pure water (18.2 MΩ cm). After being purged with argon, the solution into the electrochemical cell was kept under an argon blanket throughout the duration of the experiment. Control voltammetries were always recorded before each experiment to guarantee the cleanliness of the cell and electrolyte solution. The working electrode was always held under meniscus configuration to keep the exposed area of the electrode constant (7.1 mm^2^).

The cyclic voltammetry (CV) experiments were carried out at 25°C in an electrochemical cell with 50 mL of electrolyte consisting of 20 mM sodium phosphate buffer, pH 7.0, plus the corresponding concentration of *p*-aminophenyl β-D-galactopyranoside (PAPG, Sigma-Aldrich), a synthetic substrate of β-galactosidase that is enzymatically hydrolyzed to yield D-galactose and *p*-aminophenol (PAP). The hydrolysis of PAPG was followed by CV, monitoring the oxidation of generated PAP [40] between 0.4 and −0.2 V at 100 m V s^−1^. Voltammetries were recorded after the electrode established contact with the electrolyte solution in a meniscus configuration. In order to study the stability of the functionalized electrodes, different graphite rods were prepared simultaneously and kept at 4°C in 20 mM sodium phosphate, pH 7.0, until use. Every 24 h, a fresh solution of the chimera protein was incubated with a single modified electrode and three independent β-galactosidase activity determinations were performed by CV using PAPG as substrate. Prior to immobilization, the activity of the protein was spectrophotometrically checked using ONPG as substrate, and determined to be equivalent in all cases. Between measurements, the electrode was washed with 20 mM sodium phosphate, pH 7.0, to remove reaction products and unreacted substrate, and the cell was further washed three times with ultra-pure water, boiled once, and then the enzyme activity was determined again, so as to achieve three independent data. During the cell washing time (approximately 30 min), the electrode containing the immobilized protein was conserved submerged in 20 mM sodium phosphate buffer pH 7.0 at 4°C.

Time course experiments were carried out at 25°C in an electrochemical cell containing 10 mL of 20 mM sodium phosphate buffer pH 7.0 plus increasing concentrations of PAPG (0.1–5.0 mM). The hydrolysis of PAPG to generate PAP was monitored by registering cyclic voltamogrammes at different times and calculating the charge density. Kinetic parametres (catalytic constant –*k*_cat_– and Michaelis constant –*K*_m_) were obtained from direct fitting the charge density observed after 4 min of reaction to the Michaelis-Menten equation [41] using SigmaPlot utilities (Systat Software Inc.).

## Results and Discussion

The affinity of the C-LytA module for choline structural analogs, both in solution and as part of solid supports [6], [20], [21], [23], led us to evaluate the binding and electrochemical behaviour of a C-LytA-tagged *E. coli* β-galactosidase (CLytA-βGal) to graphite electrodes coated with a mixed self-assembled monolayer (SAM) covalently functionalized with DEAEA and EA. The β-galactosidase from *E. coli* was selected for this study because of the easeness of its assay, valuable interest on research and its wide biotechnological applications in various fields, including biosensors [42], [43]. Moreover, this work would allow a direct comparison with the performance of similarly functionalized gold electrodes [6]. To ensure the simplicity of the system, experiments were carried out in the absence of the activating Mg^2+^ cation. This does not prevent the protein to display its enzymatic activity, yet with different kinetic parametres compared to the metal-bound form [44], [45].

Freshly activated electrodes, prepared as explained in the experimental section (Fig. 1A), were incubated with the CLytA-βGal chimera or the untagged β-galactosidase control protein. After extensively washing the electrode with buffer containing 100 mM NaCl in order to remove non-specifically bound protein, the hydrolase activity was detected by absorption spectroscopy using ONPG as substrate, indicating a correct modification of the electrode and the specific immobilization through the affinity tag of active protein. Besides the DEAEA ligand, different concentrations of EA were included in the functionalization mix as spacers in order to reduce the steric hindrances provided by the bulky DEAEA groups [1], [2] as well as the highly positive charge in the surface that might influence both the stability of the SAM (by severe electrostatic repulsions) and the non-specific interactions with negatively charged proteins. A molar ratio 8∶1 of EA/DEAEA was found to be optimal for the best binding of CLytA-βGal, leading to the immobilization of 1.1±0.1 galactosidase units per mm^2^ of geometric electrode area. Supposing that the specific activity of the free enzyme (26 units per microgram in the absence of Mg^2+^ according to Viratelle and Yon [45]) is not affected upon immobilization, around 313 fmol of protein per mm^2^ are estimated to be adsorbed onto the electrode, a value that is almost 14-fold higher than that previously reported for the same chimeric protein on gold electrodes [6] (23 fmol mm^−2^). This difference may be explained by the microstructure of graphite electrodes, which present a roughness degree that increases the effective interface area with the solution (as compared to the theoretical geometrical area), thus facilitating the adsorption of a higher load of enzyme.

**Figure 2:**
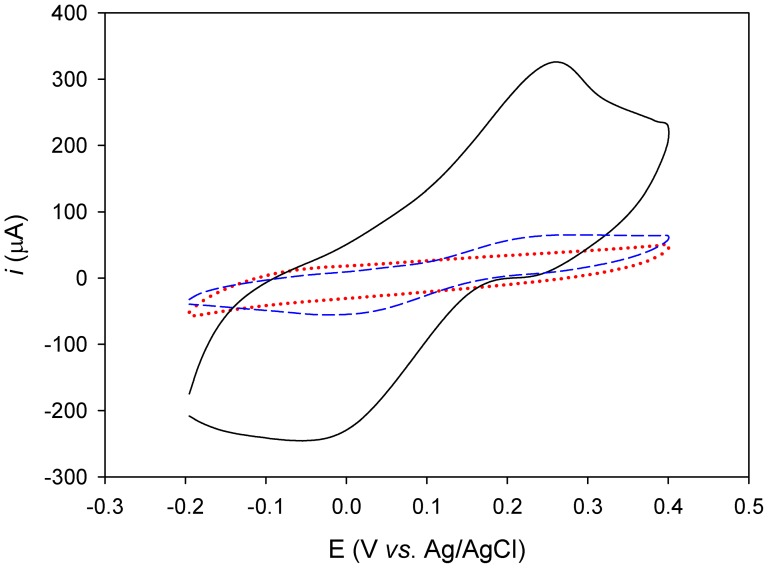
Galactosidase activity of the CLytA-βGal hybrid protein anchored onto DEAEA/EA-modified graphite electrodes at 25°C. The product of the catalytic reaction, 4-aminophenol (PAP), was followed by cyclic voltammetry (solid line), starting in the anodic direction at −0.2 V and covering a potential range from 0.4 to −0.2 V at 100 mV s^−1^. The electrolyte contained 20 mM sodium phosphate, pH 7.0, plus 4 mM of PAPG as enzymatic substrate. Voltammograms were recorded 6 minutes after being in contact the protein with the electrolyte. Potentials are quoted versus the Ag/AgCl, 3 M KCl electrode. Control experiments without enzyme (red dotted line) and with untagged β-galactosidase (blue dashed line) are also depicted.

Once determined the optimal conditions for CLytA-βGal binding to the graphite electrode, the enzymatic activity of the chimera was further checked by CV, monitoring the oxidation of PAP produced upon PAPG hydrolisis (Fig. 1B). Figure 2 shows a strong oxidation-reduction peak centred at around 0.1 V, representative of PAP accumulation on the electrode surface as a consequence of the immobilized CLytA-βGal activity. As a comparison, untagged β-galactosidase only yielded a very small PAP signal accounting for less than 3% of that produced by the chimera (Fig. 2), indicating that the residual interaction of non-fused β-galactosidase with the modified electrode is minimal. These results therefore indicate that the CLytA-βGal hybrid protein specifically binds to the electrode as oriented by the C-LytA module and maintains its catalytic activity.

**Figure 3:**
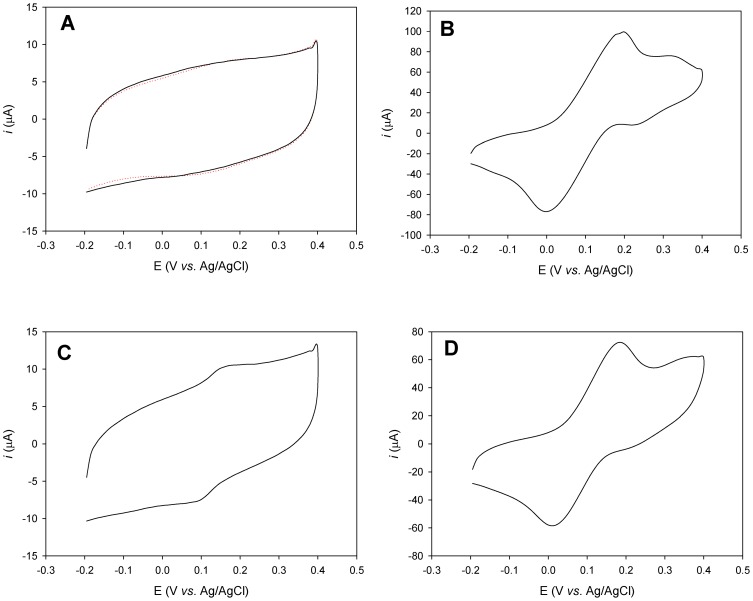
Enzymatic activity of modified graphite electrodes incubated with CLyt-βGal in phosphate buffer with different additions. (A) Immobilization of CLyt-βGal in the presence of 1 M choline. Dotted line depicts the control experiment without enzyme. (B) Immobilization of CLyt-βGal in the presence of 1 M NaCl. (C) Electrode used in (B), after 10 min incubation in 1 M choline solution. (D) Electrode used in (C), after washing with 1 M NaCl and subsequent immersion in a fresh solution of CLyt-βGal in the presence of 1 M NaCl. Cyclic voltammograms at 100 m V s^−1^ were recorded 10 min after the addition of 2.5 mM PAPG. Potentials are quoted versus the Ag/AgCl, 3 M KCl electrode.

Further evidence for the specificity of the tagged protein adsorption is provided in Figure 3. No PAPG hydrolysis was detected if the CLyt-βGal immobilization was carried out in a solution containing 1 M choline (Fig. 3A), most probably due to competition of the free ligand with the DEAEA residues in the electrode for the choline-binding sities of the protein. Non-specific ionic strength effects can be ruled out, as the usual PAP signal was recorded in the presence of 1 M NaCl in the protein incubation solution (Fig. 3B). Moreover, the ability of the latter enzymatic electrode to hydrolyze PAPG was lost upon incubation with 1 M choline solution (Fig. 3C), which elutes most of the protein from the electrode. Furthermore, the signal was restored again (approximately 80% of the initial oxidation charge signal) upon NaCl washing and reloading with fresh protein (Fig. 3D), a comparable result with that of gold-immobilized enzyme [6]. The remaining PAP production detected after choline washing (Fig. 3C) is consistent with the results presented in Figure 2, and suggests residual adsorbed protein through scarce non-specific interactions that are not weakened by choline or NaCl, as also observed by Madoz and coworkers [6]. All the data together demonstrate that the immobilization of the tagged protein is specific and strong but reversible, and that the anchoring process does not inhibit the catalytic activity of the protein.

**Figure 4:**
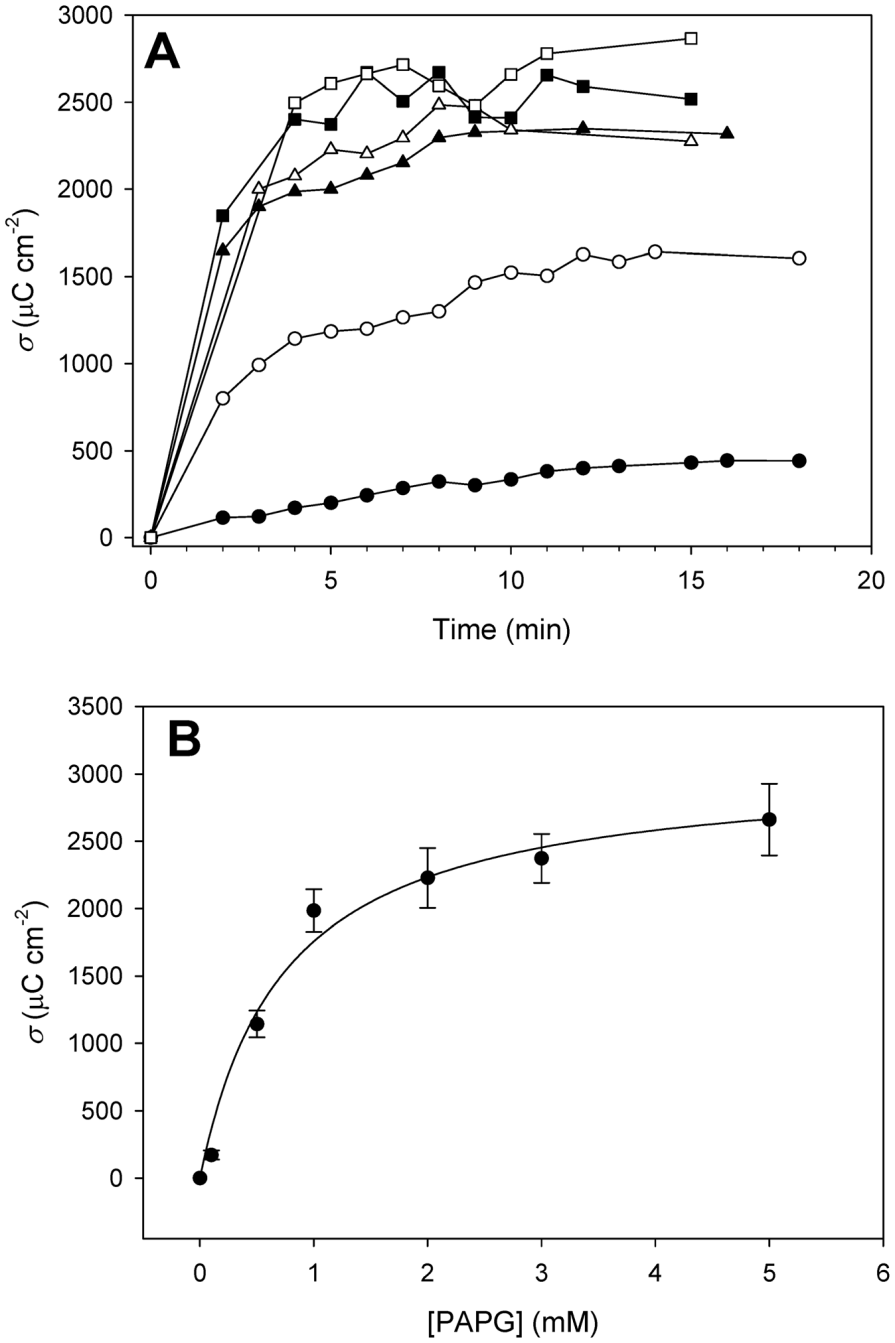
Kinetic properties of immobilized CLyt-βGal. (A) Voltammetric detection of PAP as a result of PAPG hydrolysis by CLyt-βGal immobilized on functionalized-graphite electrodes. The electrolyte contained 20 mM sodium phosphate buffer pH 7.0, plus increasing concentrations of PAPG. The oxidation charge density is represented considering the geometric surface area of the electrode (7.1 mm^2^). (B) Representation of the oxidation charge density registered after 4 min of incubation under the experimental conditions in (A), and fitting to the Michaelis-Menten equation. The results are the average of three experiments, and error bars have been ommited from panel (A) for the sake of clarity, although they are represented in Figure S1.

In order to get a deeper insight into the enzymatic activity of the immobilized fusion protein, its kinetic characteristics were evaluated by voltammetric detection of PAP at increasing concentrations of PAPG. As shown in Figure 4A and Figure S1, the oxidation charge density increases with time, yielding typical saturation kinetics at each substrate concentration. The general response of the immobilized enzyme after 4 min of incubation at different concentrations of PAPG was adjusted to a Michaelis-Menten equation (Fig. 4B), yielding a maximum charge transfer after that time of 3061±189 µC cm^−2^, corresponding to a catalytic constant (*k*_cat_) of 4.3±0.3 s^−1^ and a *K*_m_ value of 0.8±0.1 mM. The *k*_cat_ value is 20-fold lower than that of CLyt-βGal immobilized in gold electrodes [6] (86 s^−1^), whereas the Michaelis constant is 2 times higher than the gold electrode-immobilized protein (0.4 mM) and about 2.5-fold larger than that reported for untagged β-galactosidase in solution [45] (33 mM). Nevertheless, it should be remarked that in these two latter cases the activating Mg^2+^ ion was included in the solution, a fact that does increase the *k*_cat_ and decrease the *K*_m_ values in about 15-fold and 4-fold respectively at least for the closely related ONPG substrate [44], [45].

**Figure 5:**
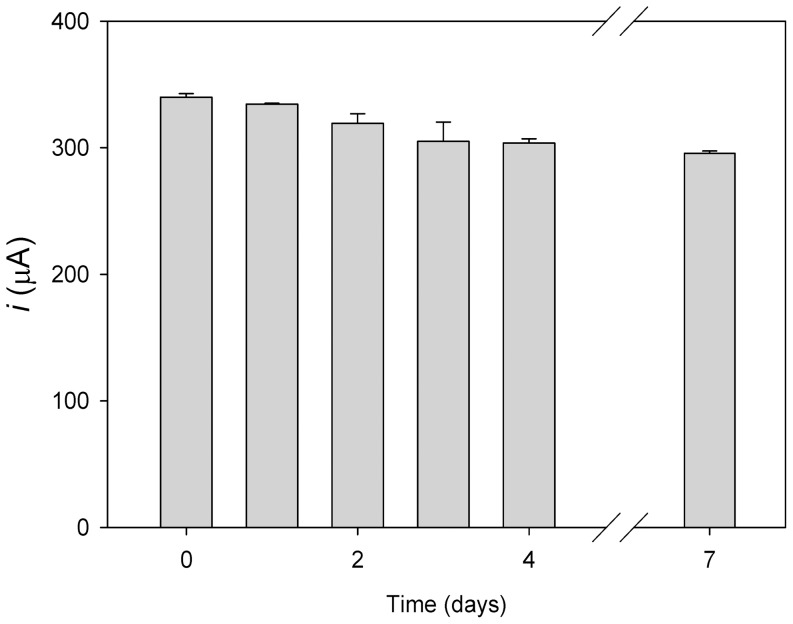
Evaluation of the stability of the SAM and the recycling of the modified electrode. CLyt-βGal was immersed on functionalized graphite electrodes stored at 4°C for the indicated time, and the catalytic activity of immobilized enzyme was evaluated with 4 mM PAPG. Voltammograms were recorded 6 min after being in contact the electrode with the substrate, at 100 mV s^−1^. After each measurement the electrode was washed with 20 mM sodium phosphate, pH 7.0 plus 100 mM NaCl and checked for remaining activity. The results are the average of three experiments in which the immobilization and enzymatic measurements were repeated independently.

One of the key points related to enzymatic biosensors development require the evaluation of device reproducibility and the capacity of system reutilization, an aspect that strongly depends on the stability of the functionalized surface. Although the modification of the electrode surface by the reductive adsorption of the APA diazonium salt generates a covalent C-C bond with the carbon surface significantly stronger than others, as for example the gold-thiol bond [29], it was necessary to check the stability of the DEAEA/EA activated SAM on the electrode. To do so, we studied the binding capability of different electrodes simultaneously functionalized and conserved at 4°C, with time. Every 24 h, one of the electrodes was incubated with freshly purified fusion protein and, after washing, its binding to the electrode was evaluated by CV detection of the PAP accumulated due to the PAPG hydrolisis. Figure 5 shows that after 7 days incubation at 4°C the initial β-galactosidase activity was retained in a 87%, indicating that the modification of the graphite electrodes performed is stable and maintains the full ability to bind the choline-binding domain. In contrast, when an electrode containing the immobilized fusion protein was stored in phosphate buffer at 4°C, a half-life of 3 days was determined (data not shown). This should be ascribed most probably to a gradual loss of β-galactosidase activity upon storage, especially in the absence of magnesium ion [46], and/or protein desorption rather than SAM instability, as shown above, as the isolated C-LytA module has been shown to be retained on similar funtionalized surfaces for time periods longer than 4 weeks (data not shown).

## Conclusions

We have presented a simple and cost-effective procedure for the covalent functionalization of graphite electrodes with choline analogs in order to demonstrate the feasibility of the C-LytA choline-binding module as an affinity tag to achieve the specific immobilization of recombinant proteins on the modified electrodes. Our results demonstrate that the synthetic route developed here, making use of diazonium chemistry, produces stable functionalized SAMs, which are able to reversibly bind proteins fused to the affinity tag C-LytA in an strong and specific manner and simple enough to be performed in laboratories not directly involved in organic synthesis. Our approach expands the applicability of the C-LytA tag for the immobilization of recombinant proteins on any material susceptible to be funcionalized by diazonium chemistry, such as carbon-based substrates (graphite, graphene or carbon nanotubes), and with the final aim of constructing enzymatic electrochemical cells and biosensors that can be easily regenerated and reused after the eventual enzyme inactivation simply by washing the electrode with choline and reloading with a fresh protein preparation. The C-LytA system presents some advantages over other affinity tags, such as a higher buffer compatibility, non interference with metalloproteins, a complete reversibility of binding (allowing the reusability of the electrode) and, above all, the possibility of employing tailor-made supports (in this case, carbon-based) with a wide range of compounds to choose from (tertiary and quaternary alkyl amines, all of them ligands of C-LytA [20], [21]) using simple diazonium chemistry.

## Supporting Information

Figure S1 **Voltammetric detection of PAP generated by immobilized CLyt-βGal.** Data are the same as in Figure 4, but displaying the corresponding error bars.
(PDF)
