# Supplementary material for: Specific and Reversible Immobilization of Proteins Tagged to the Affinity Polypeptide C-LytA on Functionalized Graphite Electrodes
Source: PLoS One. 2014 Jan 31;9(1):e87995. doi: 10.1371/journal.pone.0087995 (PMC3909327; doi:10.1371/journal.pone.0087995)
Supplement: Figure S1 — Voltammetric detection of PAP generated by immobilized CLyt-βGal. Data are the same as in Figure 4, but displaying the corresponding error bars. (PDF) [file pone.0087995.s001.pdf]

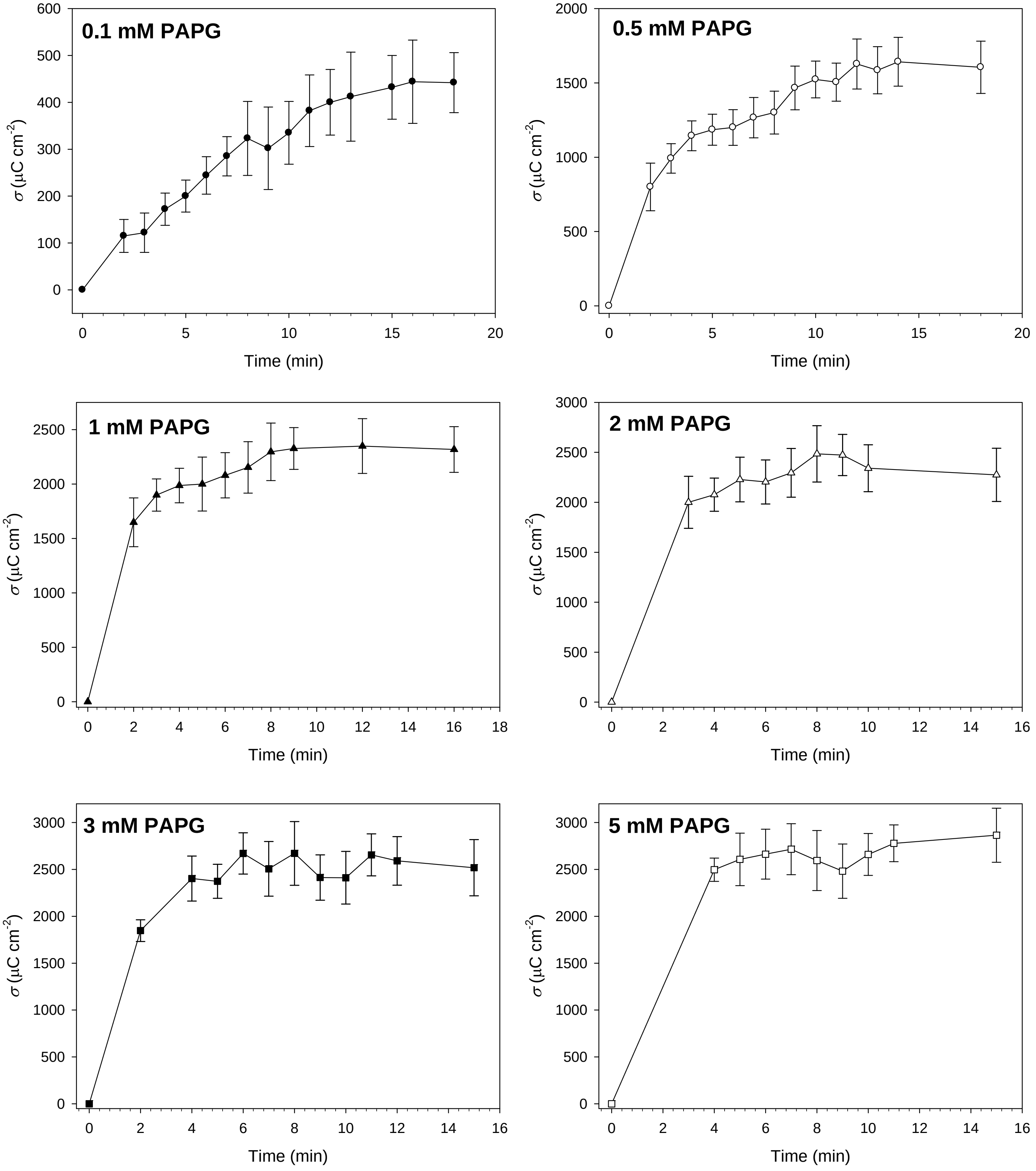

Supplementary Figure S1. Voltammetric detection of PAP generated by immobilized CLyt-βGal. Data are the same as in Figure 4, but displaying the corresponding error bars.
